# Development of eHOME, a Mobile Instrument for Reporting, Monitoring, and Consulting Drug-Related Problems in Home Care: Human-Centered Design Study

**DOI:** 10.2196/humanfactors.8319

**Published:** 2018-03-07

**Authors:** Nienke Elske Dijkstra, Carolina Geertruida Maria Sino, Eibert Rob Heerdink, Marieke Joanna Schuurmans

**Affiliations:** ^1^ Research Group Care for the Chronically Ill University of Applied Sciences Utrecht Utrecht Netherlands; ^2^ Research group Innovation in Pharmaceutical Care University of Applied Sciences Utrecht Utrecht Netherlands; ^3^ Division of Pharmacoepidemiology and Clinical Pharmacology Utrecht Institute for Pharmaceutical Sciences Utrecht Netherlands; ^4^ Julius Center for Health Sciences and Primary Care Department of General Practice University Medical Center Utrecht Utrecht Netherlands

**Keywords:** primary care, home care, eHealth, mHealth

## Abstract

**Background:**

Home care patients often use many medications and are prone to drug-related problems (DRPs). For the management of problems related to drug use, home care could add to the multidisciplinary expertise of general practitioners (GPs) and pharmacists. The home care observation of medication-related problems by home care employees (HOME)-instrument is paper-based and assists home care workers in reporting potential DRPs. To facilitate the multiprofessional consultation, a digital report of DRPs from the HOME-instrument and digital monitoring and consulting of DRPs between home care and general practices and pharmacies is desired.

**Objective:**

The objective of this study was to develop an electronic HOME system (eHOME), a mobile version of the HOME-instrument that includes a monitoring and a consulting system for primary care.

**Methods:**

The development phase of the Medical Research Council (MRC) framework was followed in which an iterative human-centered design (HCD) approach was applied. The approach involved a Delphi round for the context of use and user requirements analysis of the digital HOME-instrument and the monitoring and consulting system followed by 2 series of pilots for testing the usability and redesign.

**Results:**

By using an iterative design approach and by involving home care workers, GPs, and pharmacists throughout the process as informants, design partners, and testers, important aspects that were crucial for system realization and user acceptance were revealed. Through the report webpage interface, which includes the adjusted content of the HOME-instrument and added home care practice–based problems, home care workers can digitally report observed DRPs. Furthermore, it was found that the monitoring and consulting webpage interfaces enable digital consultation between home care and general practices and pharmacies. The webpages were considered convenient, clear, easy, and usable.

**Conclusions:**

By employing an HCD approach, the eHOME-instrument was found to be an easy-to-use system. The systematic approach promises a valuable contribution for the future development of digital mobile systems of paper-based tools.

## Introduction

### Background

Pharmacotherapy is one of the most common interventions used in health care. Its use has considerably grown because of the aging population and the increased prevalence of chronic diseases [1]. Although medication may contribute to cure, slow progression, or reduce the symptoms of diseases, it is also associated with drug-related problems (DRPs). According to the Pharmaceutical Care Network Europe (PCNE), a DRP is defined as an event or circumstance involving drug therapy that actually or potentially interferes with desired health outcomes [2]. DRPs may negatively affect a person’s perceived quality of life, and it may increase morbidity, mortality, health care costs, and the risk of hospital (re)admissions [3-6]. Older people are more prone to DRPs because of the higher prevalence of drug use and age-related pathophysiologic changes in pharmacokinetics and pharmacodynamics [3,7,8]. To prevent or limit complications of DRPs in older people, a multidisciplinary approach in primary care through home care, general practices, and pharmacies is desirable [9,10]. Home care workers have insight into the home environment of the patients and can offer an important contribution to the recognition of problems related to drug use. Several tools have been developed for home care workers for the recognition of signs and symptoms of DRPs in home care patients [11-15]. One of these instruments is the validated home care observation of medication-related problems by home care employees (HOME)-instrument [15], an observation list with 28 signs and symptoms of potential DRPs categorized in 3 categories (process, pill, and patient). An observational study of the HOME-instrument by our research group [15] showed that almost half of all the observed signs and symptoms were assessed as potentially drug related. The challenges of the HOME-instrument observation forms include issues such as problems to transfer or store forms into an electronic patient file and difficulty in monitoring and comparing the progression of DRPs over time. Furthermore, it does not offer consultation with other primary care disciplines, such as general practitioners (GPs) and pharmacists. Digital health care technologies (eHealth) combining information and communication technologies may eliminate these challenges. GPs and pharmacists expressed the demand for collaboration in recognizing and managing of DRPs by using the HOME-instrument as a mobile system. Collaboration between different health care professionals can be used to develop a usable electronic HOME system (eHOME) that combines the report of DRPs (based on the content of the HOME-instrument) and the monitoring and multidisciplinary consultation of DRPs in primary care.

### Objective

This study aimed to develop eHOME, a mobile version of the HOME-instrument that includes a monitoring and consulting system for primary care.

## Methods

### Design

The development of eHOME was guided by the development phase of the framework for the development and evaluation of complex interventions of the Medical Research Council (MRC) [16]. The users and stakeholders were involved throughout the development phase by means of the human-centered design (HCD) for interactive systems [17] to develop a usable eHOME-instrument that fits the needs of the end users. The study was approved by the Medical Ethics Research Committee of the University Medical Center, Utrecht (the Netherlands) (11-129/C).

### Setting and Procedure

This study was performed from September 2014 to March 2017 in a setting of home care teams, general practices, and pharmacies.

The HCD approach was divided into 6 phases: (1) the Delphi round; (2) the development, evaluation, and redesign of the prototype report webpage and the monitoring webpage interfaces for home care; (3) the usability evaluation pilot of the report webpage and the monitoring webpage interfaces for home care; (4) the expansion and development of the monitoring and consulting webpage interfaces for home care, GPs, and pharmacists; (5) the usability evaluation pilot of the report webpage and the monitoring and consulting webpage interfaces; and (6) the development of the final webpages interfaces. Table 1 shows how the 6 phases are mapped into the HCD described in ISO 92410-201 [17].

#### Delphi Round

In October 2014, a Delphi round was conducted in a workgroup of 13 participants (a postdoctoral researcher, a project manager, 2 pharmacists, 7 home care workers of one home care team, and 2 software developers). In the Delphi round, the context in which eHOME should be used and the requirements of the users for a report system (to report DRPs by home care workers who perform home visits), a monitoring and consulting system (for home care nurses, GPs, and pharmacists) and requirements on the organizational level were identified. During both parts of the round, the project manager played the role of an observer and reported the requirements on a whiteboard.

**Table 1:** Methodological phases mapped into a human-centered design (HCD). The ✓ symbol shows which phases of this study belong to which phases of the HCD.

Methodological phases			HCD^a^ phases				
			Understand and specify the context of use	Specify the user requirements	Produce design solution to meet the user requirements	Evaluate the designs against requirements	Designed solution meets user requirements
The Delphi round			✓	✓			
**The development, evaluation, and redesign of the prototype report webpage and the monitoring webpage interfaces for home care**					✓		
	Evaluation workgroup meeting					✓	
	Redesign report webpage and monitoring webpage interfaces for home care				✓		
**The usability evaluation pilot of the report webpage and the monitoring webpage interfaces for home care**						✓	
	Questionnaires					✓	
	Semistructured interviews					✓	
The expansion and development of the monitoring and consulting webpage interfaces for home care, GPs^b^, and pharmacists					✓		
The usability evaluation pilot of the report webpage and the monitoring and consulting webpage interfaces						✓	
The development of the final webpage interfaces							✓

^a^HCD: human-centered design.

^b^GPs: general practitioners.

#### Development, Evaluation, and Redesign of the Prototype Report Webpage and the Monitoring Webpage Interfaces for Home Care

Two software developers developed a prototype of the report webpage and the monitoring webpage interfaces and a link between these 2 interfaces, based on the formulated requirements of phase one. In a second workgroup meeting, the prototypes of the webpage interfaces were presented and installed on the mobile phones and personal computers of the workgroup members, and they were tested by the home care workers. The report webpage interface was tested by the home health care assistants, who performed home visits, in a case study exercise. Several home care practice-based signs and symptoms of DRPs were reported by the report webpage interface in the exercise. The home care nurse evaluated the usability of the monitoring webpage interface for the home care nurses and assessed whether the reported problems of the home health care assistants were forwarded from the report webpage to this webpage interface. This process allowed the home care workers to get used to the webpage interfaces for the usability evaluation pilot. Additionally, the workgroup meeting resulted in a plenary discussion with a set of adjustments to improve the usability of the prototype webpage interfaces. By the adjustments as a result of the plenary discussion, the content of the signs and symptoms of the report webpage for the home care was redesigned. This phase was carried out from September to October 2014.

#### Usability Evaluation Pilot of the Report Webpage and the Monitoring Webpage Interfaces for Home Care

To assess the usability of the webpage interfaces, a usability evaluation pilot among the home care team was performed. During a 6-week pilot period (November to December 2014), the home health care assistants (n=6), who performed home visits, reported signs and symptoms of DRPs by the report webpage interface. At the end of the pilot period, the home health care assistants answered a questionnaire about the usability of the report webpage interface. To explore the usability of the monitoring webpage, a semistructured interview with the home care nurse was carried out. The interview was audiotaped and transcribed verbatim. Data of the questionnaires and the semistructured interview resulted in a set of requirements to improve the usability of eHOME.

#### Expansion and Development of the Monitoring and Consulting Webpage Interfaces for Home Care, General Practitioners, and Pharmacists

Between January and May 2015, the software developers expanded the monitoring webpage interface for the home care nurse with a consulting system. Furthermore, the monitoring and consulting webpage interfaces for the GPs and the pharmacists and a link between the monitoring and consulting webpage interfaces that allows 2-way communication between the home care nurse and the GP or the pharmacist was developed.

#### Usability Evaluation Pilot of the Report Webpage and the Monitoring and Consulting Webpage Interfaces

To test the possibility of communication between the home care nurses and the GPs or the pharmacists through the monitoring and consulting webpage interfaces, a second evaluation pilot of 3 months was initiated. Using a convenience sample strategy, 2 groups were selected. The first group consisted of 12 home care teams and 3 pharmacies. The pilot of group one took place from November 2015 to February 2016. The second group consisted of 6 home care teams, 7 general practices, and 6 pharmacies. The pilot in the second group took place from March to June 2016. Before the start of the pilot, participants received information about the types and the consequences of DRPs in home care patients, the importance of early recognition of DRPs, the webpage interfaces, and an explanation of the pilot in a workshop meeting. The workshop was led by a home care nurse, 2 pharmacists, and the first author (ND). During both the pilots, the home care workers, who performed home visits, reported the signs and symptoms of DRPs by the report webpage interface, and the home care nurses performed daily triage of the reported signs and symptoms with the monitoring and consulting system. Nurses in group one could report problems to a pharmacist and nurses in group two could report problems to a GP and or a pharmacist by using the monitoring and consulting webpage interfaces. The GP or the pharmacist was asked to respond to the signs and symptoms by the monitoring and consulting webpage interfaces. Anonymized descriptive statistics of consultation data between home care nurses, GPs, and pharmacies showed whether the consultation between the users occurred. Semistructured interviews with 8 home care workers were performed following the pilot. The interviews were held at the home care workers’ home or the home care practice. Besides the interviews, every 2 weeks, an evaluation with the home care nurses (n=18) through a phone call was carried out. The interviews and the evaluation with the home care nurses aimed to explore the strengths and the weaknesses of the webpage interfaces and to explore the potential solutions. The interviews and biweekly evaluations were carried out by the home care nurse of the workgroup and ND. The interviews were audiotaped and transcribed verbatim.

#### Development of the Final Webpages Interfaces

Data of the semistructured interviews and the biweekly evaluations were used to adjust the webpage interfaces so that a final version of the webpage interfaces that meets the user requirements could be designed. The final version of the webpage interfaces was developed by the software developers between October 2016 and February 2017.

## Results

The results are described for the following 6 phases: (1) the Delphi round; (2) the development, evaluation, and redesign of the prototype report webpage and the monitoring webpage interfaces for home care; (3) the usability evaluation pilot of the report webpage and the monitoring webpage interfaces for home care; (4) the expansion and development of the monitoring and consulting webpage interfaces for home care, GPs, and pharmacists; (5) the usability evaluation pilot of the report webpage and the monitoring and consulting webpage interfaces; and (6) the development of the final webpages interfaces.

### Delphi Round

#### Context of Use

To achieve optimal management of DRPs by eHOME, it was determined that eHOME should be used by a multidisciplinary team of home care workers, GPs, and pharmacists. The goal for optimal management of signs and symptoms of DRPs in home care patients by eHOME is twofold. First, home health care assistants who visit clients for essential care (activities of daily living such as bathing) report the signs and symptoms. Second, a bachelor trained home care nurse performs a daily triage of the reported signs and symptoms and forwards problems to a GP or a pharmacist when their expertise is needed. Subsequently, the GP and pharmacist send feedback on the DRPs to the home care nurse. It was decided that eHOME must consist of 2 webpage interfaces: a report webpage interface for home health care assistants who perform home visits, and monitoring and consulting webpage interfaces for home care nurses, general practices, and pharmacies.

#### User Requirements eHOME

Requirements for both webpage interfaces and requirements on the organizational level were formulated.

#### Report Webpage Interface

The content of HOME-instrument [15] formed the basis for the report webpage interface. Home care workers unanimously decided that the report webpage interface needs to include the signs and symptoms of the HOME-instrument, 6 common home care practice-based problems: (1) medication used has not been listed on the medication list; (2) medication on the medication list is not in use; (3) home care patient uses other amounts or dosages; (4) home care patient uses medication on another time (notice which medicines and when); (5) thick legs or feet; and (6) wounds, and a possibility to add any other potential sign or symptom of a DRP. To monitor DRPs for a longer period (eg, thick legs or feet or bruises), the possibility to add photos of these observations was required.

#### Monitoring and Consulting Webpage Interfaces

The monitoring and consulting webpage interfaces must contain the reported DRPs of home care patients out of the report webpage interface (with information of client’s name, sex, and date of birth), possibility to add new profiles of home care patients with detailed identification details (eg, name[s], sex, date of birth, name of GP, and pharmacist).

#### Organization Level

A link between the report webpage and the monitoring and consulting webpage interfaces of the home care and a link between the monitoring and consulting webpage interfaces of home care nurses and GPs and pharmacists was required so that the information of reported problems between the webpage interfaces could be shared. Furthermore, the webpage interfaces needed to be available for smartphones, tablets with the iOS and Android operating systems, as well as personal computers. All webpage interfaces must contain a log-in screen, so personal details of patients could be assessed by the users only by entering a username and a password in the log-in screen.

### Development, Evaluation, and Redesign of the Prototype Report Webpage and the Monitoring Webpage Interfaces for Home Care

One report webpage interface was developed in which the DRPs could be reported and forwarded to the monitoring webpage of the home care nurse. For the home care nurse, one monitoring webpage was developed for incoming DRPs. All home care workers were able to report the problems of the case study exercise by the report webpage interface. Additionally, it was found that the reported problems appeared all in the monitoring webpage interface. Following the evaluation pilot, it was decided that the content of the first version of the report webpage (presented in Multimedia Appendix 1) needed to be divided into several webpages to improve the usability. Instead of one report webpage, including 33 signs and symptoms, 10 webpages were developed. The 33 signs and symptoms were replaced into 2 main categories (represented with an icon) and 7 subcategories. Furthermore, some textual changes were made to enhance the readability. Specification of the changes and the final content of the report webpage interfaces are presented in Multimedia Appendix 2.

### Usability Evaluation Pilot of the Report Webpage and the Monitoring Webpage Interfaces for Home Care

Seven home care workers of the workgroup took part in the first pilot. Home health care assistants, who performed the home visits (n=6), indicated that the report webpage interface is a convenient, clear, and a usable instrument. One home health care assistant missed the possibility to review their own reported observations, and 3 home health care assistants reported the need for a conformation that an observation was forwarded to the home care nurse. The home care nurse commented in the interview adjustments to optimize the monitoring webpage interface; first, a link between the electronic patient file and the monitoring webpage interface was mentioned so that patient data will automatically be added to the webpage interfaces. Thereby adding a client profile manually to the monitoring webpage can be avoided which is time-saving. Second, the possibility to forward observations of signs and symptoms to a GP and a pharmacist was desired so that phone-calls can be avoided, which is in turn time-saving.

### Expansion and Development of the Monitoring and Consulting Webpage Interfaces for Home Care, General Practitioners, and Pharmacists

As a result of the usability evaluation pilot, it was possible to expand the monitoring webpage for the home care nurse with a consulting service and to develop a monitoring and consulting webpage for GPs and pharmacists and a link between the monitoring and consulting webpage of the home care nurse and the GPs and pharmacists.

### Usability Evaluation Pilot of the Report Webpage and the Monitoring and Consulting Webpage Interfaces

Home health care assistants indicated in interviews that they were more aware of the problems related to medication use because of the signs and symptoms presented in the report webpage. Furthermore, the report webpage was considered to be easy to use, and specific problems of the various categories of medication problems and body symptoms were usable and easy to find. Home health care assistants were able to add a note and a photo, but only after a problem was already sent to the home care nurse. This sequence was perceived as not logical, and a reverse sequence was mentioned as a solution. The home care nurses were satisfied with the clear overview with the types and amount of problems per client of the monitoring and consulting webpage. Home care nurses indicated that by using the monitoring and consulting system service, more collaboration with pharmacists and GPs were experienced leading to more solutions for DRPs. Home care nurses informed home health care assistants about the feedback of a GP or pharmacist by a telephone call, which was experienced as time consuming. The solution to forward feedback of a home care nurse, a GP, or a pharmacist from the monitoring and consulting webpage to the report webpage was mentioned. Furthermore, home care nurses indicated that the daily triage of the report problems was necessary but not performed as intended because of workload and because they needed to get used to the daily task. Therefore, some home care nurses decided to share the triage task with nurses who had the capability to perform a triage. The home care nurses indicated that if a problem is reported during a home visit, a notification by means of a pop-up on their mobile device will ensure that daily triage will take place. Adding new client profiles in the monitoring and consulting webpage was experienced as time-consuming. During the pilot, the home care nurses decided to share this task with home health care assistants. Home care workers mentioned that a link between the electronic patient file and the monitoring and consulting webpage is desirable and will ensure that data of clients who are added to the home care will be automatically added to the monitoring and consulting webpage. Extraction data showed that communication between the home care and pharmacies in group one and communication between home care and pharmacies and between home care and general practices in group two using the monitoring and consulting webpage interfaces was possible during the pilot.

### Development of the Final Webpages Interfaces

**Figure 1:**
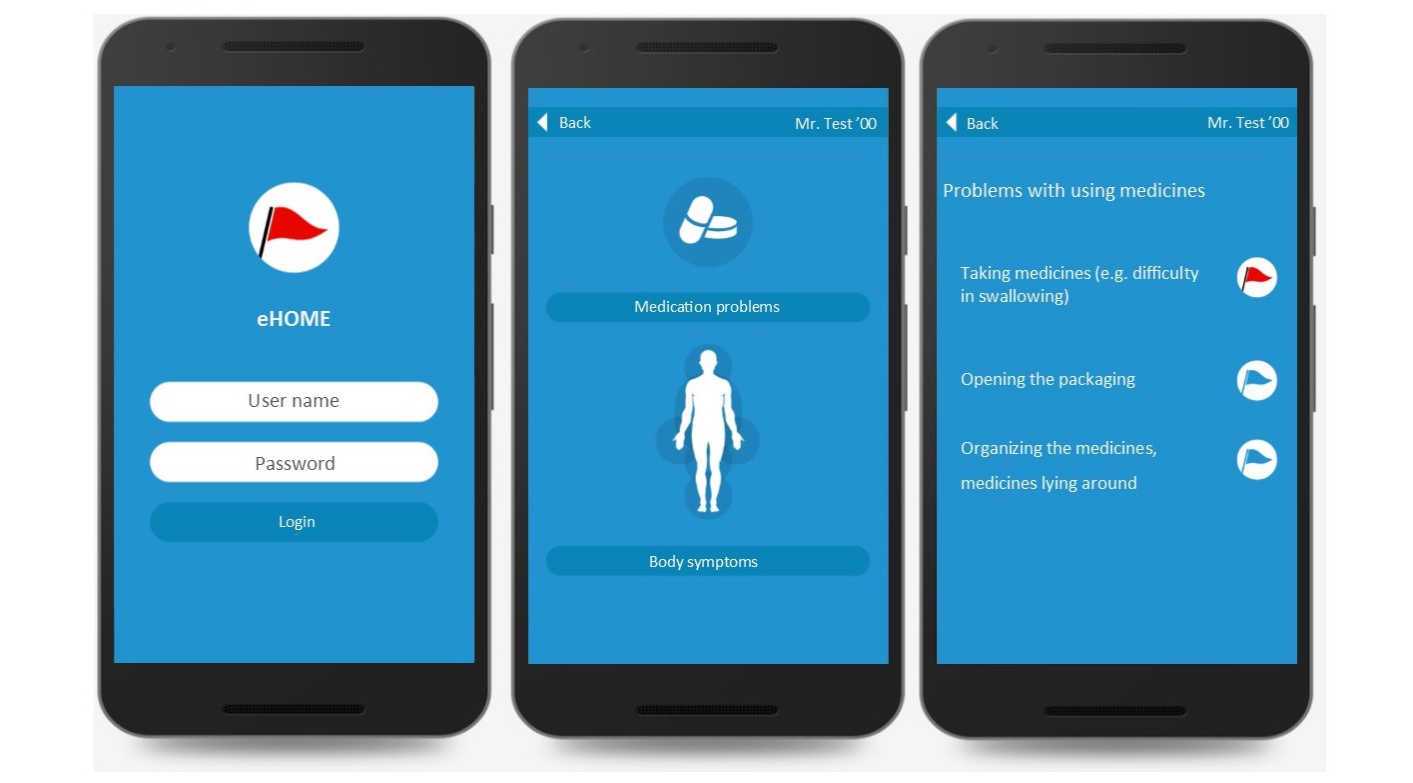
Screenshot of the report web page interface.

**Figure 2:**
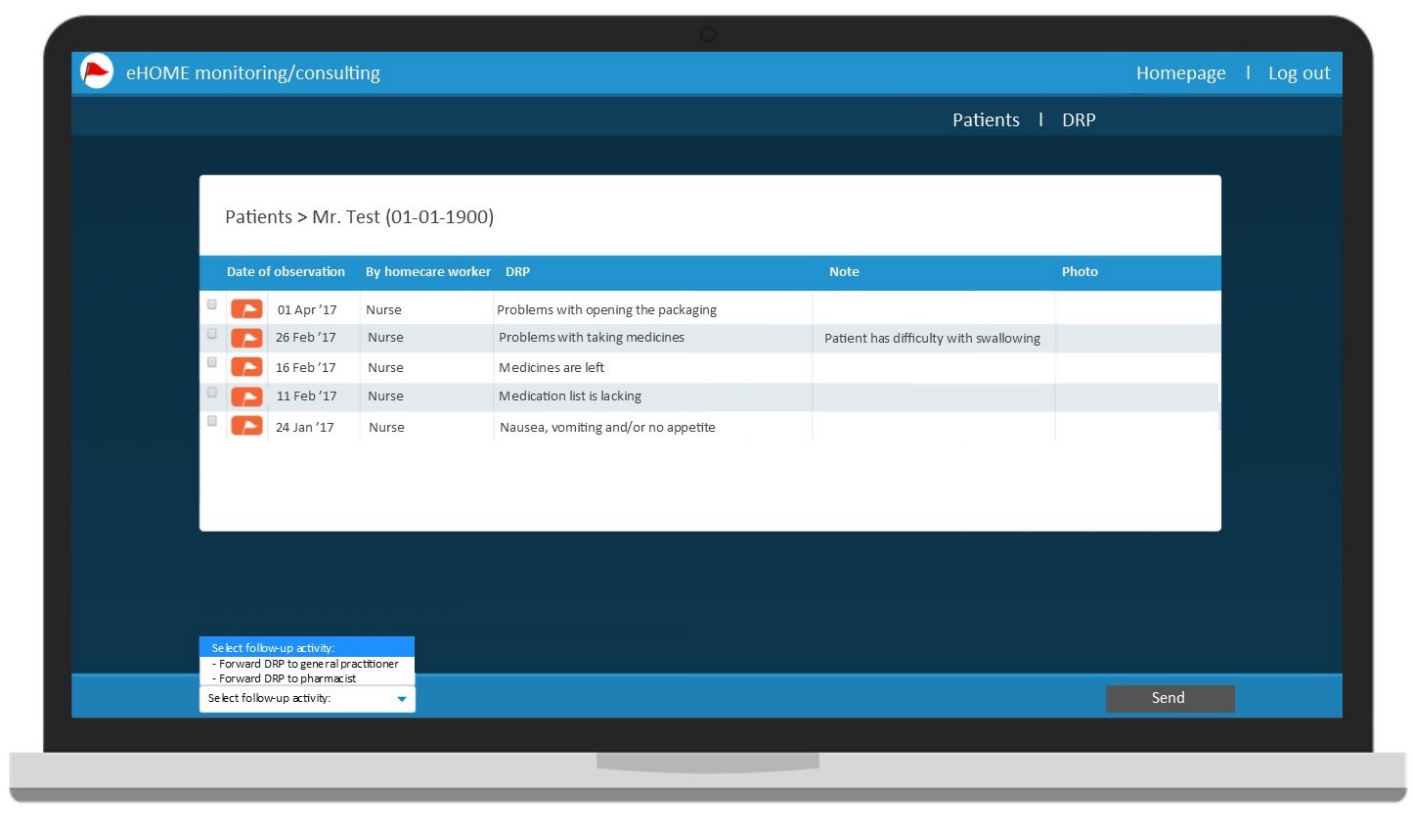
Screenshot of the monitoring and consulting web page interfaces.

In the report webpage interface, the sequence to add a note and a photo to a problem before sending the problem to the home care nurse was adjusted. Furthermore, this webpage was expanded with an extra webpage, for the feedback of home care nurses, GPs, and pharmacists on reported DRPs (see Figure 1 for screenshots of the report web page interface). The following aspects of the monitoring and consulting webpage interfaces were modified: a consulting service between the monitoring and consulting webpage interfaces of nurses and the report webpage interface of home health care assistants; a pop-up notification for incoming problems for home care nurses, general practices, and pharmacies; a link between the electronic patient files and the report webpage and the monitoring and consulting webpage for home care; and a link between the monitoring and consulting webpages of GPs and pharmacies to facilitate consultation between these disciplines (see Figure 2 for a screenshot of the monitoring and consulting web page interfaces).

## Discussion

### Principal Findings

This study resulted in eHOME, a mobile version of the HOME-instrument and a monitoring and consulting system for primary care that will help home care workers to report observed DRPs during home visits and to communicate DRPs with GPs and pharmacists.

In our study, a systematic development of the eHOME webpages was conducted. The systematic development of this study reflects the phases of an HCD therefore, the methods of this development study are mapped into the HCD approach [17]. The HCD approach process enabled collaborative decision making by home health care assistants, home care nurses, GPs, and pharmacists for the design and development of eHOME webpage interfaces, which was found to be convenient, clear, and easy to use.

### Limitations

Within the HCD approach, it is important to involve all humans (eg, users and stakeholders) from the beginning of the development process to develop a system that fits the context in which the system will be used and to meet the user requirements and technical requirements, which may increase the usability in clinical practice. In this study, the GPs, one group of users, are not involved in the context of use and user requirements analysis and the first usability evaluation pilot. Even though GPs were not involved in the decision-making process of the context of use and user requirements analysis and the first usability evaluation pilot, the second usability evaluation pilot showed that the monitoring and consulting webpage was usable for their clinical practice.

With the help of this study, the report and monitoring and consulting webpage interfaces have been developed however, the effectiveness of the multidisciplinary approach of DRPs by eHOME on patient outcomes is not yet known. Further research on the clinical effectiveness of eHOME on patient outcomes is needed.

### Comparison With Prior Work

Several paper-based report tools for DRPs are available for home care patients [11-15]; however, to our knowledge, eHOME is the first digital tool for the report of DRPs in combination with a monitoring and multidisciplinary consultation service between the home care, general practices, and pharmacies.

Previously, other paper-based screening tools were converted to mobile versions, for example, D-VAS for pain assessments [18], MOST-92610 [19], the ACEmobile [20] for assessments of neurocognitive disorders, the CVD risk assessment app [21], and the Risk detection app (in Dutch: Risico signaleren app) [22]. However, detailed information regarding the methodology used to transit from the paper screening tool to a digital system has, to our knowledge, never been published. This information is of importance to determine which different phases of the transition process lead to the usability of a system and to learn from barriers and strengths of the transition process.

This study shows how through a systematic approach a paper tool was transformed to a digital system. Developing a paper-based digital system by a systematic approach is expected to enhance the usability in clinical practice. Furthermore, the transparency of this systematic process is of importance and helps others to plan and manage methodological phases of the transition process of paper-based tools into usable digital systems, when and how health care professionals and other stakeholders can be involved, and to consider barriers and strengths of a development process.

Within this study, the focus on recognition of DRPs was for older patients by the home care workers. However, in intramural care settings, such as nursing homes and hospitals, older patients are also vulnerable for DRPs and dependent on health care professionals. Therefore, eHOME can be used by health care professionals in several care settings.

### Conclusions

By employing an HCD approach, the HOME-instrument was converted to eHOME webpage interfaces, which was considered convenient, clear, and easy to use for the report of the signs and symptoms of potential DRPs in home care patients and for the monitoring and multidisciplinary consultation of these problems in primary care. This study provides a description of a systematic approach that can be used for future development of digital systems of paper-based tools.

## Appendix

Multimedia Appendix 1 Content of the first version of the report webpage for home care.

Multimedia Appendix 2 Content of the second version of the report webpage for home care.

## Abbreviations

DRPs: drug-related problems
eHealth: electronic health
eHOME: electronic HOME
GPs: general practitioners
HCD: human-centered design
HOME-instrument: home care observation of medication-related problems by home care employees instrument
MRC: Medical Research Council
