# Supplementary material for: Development of eHOME, a Mobile Instrument for Reporting, Monitoring, and Consulting Drug-Related Problems in Home Care: Human-Centered Design Study
Source: JMIR Hum Factors. 2018 Mar 7;5(1):e10. doi: 10.2196/humanfactors.8319 (PMC5863008; doi:10.2196/humanfactors.8319)
Supplement: Multimedia Appendix 1 [file humanfactors_v5i1e10_app1.pdf]

Content of the first version of the report web page for the homecare
A Is there a printout of the list of medication from the pharmacy?<br>Does the patient take the medicines on the list?<br>Does the patient have stomach ache?<br>Does the patient have very black feces?<br>Does the patient have regular nosebleeds?<br>Does the patient have bruises / black and blue spots?<br>Does the patient have dizziness when standing up?<br>Does the patient have drowsiness?<br>Does the patient have thirst?<br>Does the patient have (increasingly more) tightness of chest?<br>Does the patient have fainting spells?<br>Does the patient have nausea, vomiting and/or no appetite?<br>Does the patient have abdominal pain and/or no bowel movement for more than 5 days?<br>For diabetes: Irregular heart rhythm and transpiration?<br>For diabetes: Feeling of hunger?<br>Has the patient fallen recently without a clear cause?<br>Does the patient regularly forget to take his/her medication<br>Does the week package contain medicine from previous days?<br>Does the robot-dispensed dosing aid contain pouches of medicine from previous days?<br>Is the supply of medicine in house disordered?<br>Is the supply of medicine in house regularly insufficient?<br>Does the patient have problems taking the medication? (for example, problems swallowing)<br>Does the patient have trouble opening the packaging?<br>Does the patient have pain?<br>Does the patient take more than 8 painkillers a day without a prescription?<br>Does the patient use other painkillers without a prescription?<br>Does the patient drink more than 3 glasses of alcohol a day?<br>Is the patient very different from usual? (for example, suddenly confused, very irritable or lethargic)<br>Other comments about the medicines
