# Supplementary material for: Development of eHOME, a Mobile Instrument for Reporting, Monitoring, and Consulting Drug-Related Problems in Home Care: Human-Centered Design Study
Source: JMIR Hum Factors. 2018 Mar 7;5(1):e10. doi: 10.2196/humanfactors.8319 (PMC5863008; doi:10.2196/humanfactors.8319)
Supplement: Multimedia Appendix 2 [file humanfactors_v5i1e10_app2.pdf]

Changes specified
Signs and symptoms were replaced into two main categories (1. medication problems and 2. body symptoms), and needed to be represented with an icon.<br><br>The main category ‘medication problems’ contains 15 signs and symptoms, which was divided into 4 subcategories:<br>1. Problems with the medication list,<br>2. Problems with the supply of medication,<br>3. Problems with using medicines and<br>4. Homecare patients also used.<br><br>The main category ‘body symptoms’ contains 18 signs and symptoms, which was divided in 3 subcategories:<br>1. Problems with the head,<br>2. Problems with torso and<br>3. Problems with feet.<br><br>Interrogative sentences are redefined in to confirming sentences, the words 'Has the patient’, ‘Does the (patient (have/take/use))’, ‘for diabetes’, and ‘Regularly’ is removed from the sentences and four sentences with specific signs/symptoms were redefined (1. 'Robot-dispensed dosing aid' in to ‘Medication pouches contains medicines of previous days’ since not all homecare patients have a robot-dispensed dosing aid, 2. 'Does the patient use other painkillers without a prescription?' is changed in to 'Homecare patient also uses other medication without prescription', 3. ‘Does the patient have bruises/black and blue spots?’ is changed in to ‘bruises’ and 4. 'Other comments about the medicines' is replaced in to 'other reports' and added to both main categories).<br><br>Furthermore 'Does the patient take the medication on the list' was removed from the content since this problem is equal to the medication problem ‘problems with the supply of medication medicines are left: forget to take medicine’.

Content of the second version of the report web pages for the homecare

Main category	Subcategory	Sign/symptoms
Medication problems [d] 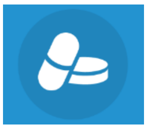	Problems with the medicine list	Medication list is lacking (medication list of the pharmacy) [a]<br>Medication used has not been listed on the medication list [c]<br>Medication on the medication list is not in use [c]<br>Homecare patient use other amounts or doses [c]<br>Homecare patient uses medication on another time (notice which medicines and when) [c]
	Problems with the supply of medication	Medication pouches contains medicines of previous days [b]<br>Week package contains medicines from previous days [a]<br>Medicines are left, forget to take medicines [a]<br>Client has insufficient medicines at home [a]
	Problems with using medicines	Taking medicines (e.g. difficulty in swallowing) [a]<br>Opening the packaging [a]<br>Organizing the medicines, medicine lying around [a]
	Homecare patient also uses	More than 8 painkillers a day without a prescription [a]<br>Other medication without prescription [b]<br>More than 3 glasses of alcohol a day [a]
	Other reports [b]	
Body symptoms [d] 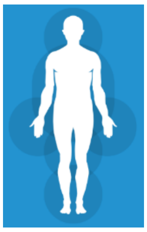	Problems with the head	Nosebleeds [a]<br>Thirst [a]<br>Drowsiness [a]<br>Tightness [a]<br>Dizziness when standing up [a]<br>Fainting spells [a]<br>Nausea, vomiting and/or no appetite [a]<br>Patient acts different from usual (e.g. extremely confuses/irritable or lethargic) [a]<br>Bruises [b]<br>Pain [c]<br>Other reports [b]
	Problems with the torso	Irregular heart rhythm and transpiration [b]<br>Stomach ache [a]<br>Feeling of hunger [b]<br>Abdominal pain and/or no bowel movement for more than 5 days [a]<br>Black feces [a]<br>Bruises [b]<br>Pain [c]<br>Other reports [b]
	Problems with feet	Fallen without a clear cause [a]<br>Thick legs/feet [c]<br>Wounds [c]<br>Bruises [b]<br>Pain [c]<br>Other reports [b]

[a] Redefined sentences
[b] One of the six sentences with redefined signs/symptoms
[c] One of the six homecare practice-based problems that have been added
[d] Added icons
